# Prevalence, patterns, functional disability of Bertolotti syndrome among patients with low back pain at Mulago National Referral Hospital

**DOI:** 10.4314/ahs.v22i4.20

**Published:** 2022-12

**Authors:** Nicholas Owen Alinda, Rodney Mugarura, Joseph Malagala, Harriet Kisembo

**Affiliations:** 1 Kumi hospital Ongino; 2 Department of Orthopaedic Surgery, Mulago National Referral Hospital; 3 College of Health Sciences Makerere University; 4 Department of Radiology, Mulago National Referral Hospital

**Keywords:** Bertolotti syndrome, Transitional vertebrae, Functional disability

## Abstract

**Background:**

Bertolotti syndrome is a differential diagnosis in back pain. We know little about it in Uganda. This study aimed to describe the prevalence, clinical and radiological patterns of Bertolotti syndrome and functional disability associated with it.

**Methods:**

We did a descriptive cross-sectional study at the spine outpatients' clinic of Mulago National Referral Hospital. We screened patients with chronic low back pain for lumbosacral transitional vertebrae over four months and classified them according to Castellvi. We collected demographics, clinical symptoms, and functional disability data and summarized it descriptively.

**Results:**

Out of 385 patients, we identified 39 with Bertolotti syndrome. The prevalence and the median age were at 10.1% and 49 years respectively, with most patients being females (66.7%) in the age range of (36 to 50) years, the pain started during the age range of 31–40. The commonest and least were type IIA (20.5%) and type IV (10.3%), respectively. Most patients (66.3%) had radicular symptoms, mainly the toe extension nerve root. The average visual analog scale was 6.3. However, most patients suffered from mild- to moderate disability (66.7%).

**Conclusion:**

Bertolotti syndrome is common and functionally debilitating. We should consider it in the differential diagnosis of chronic low back pain.

## Introduction

Bertolotti syndrome (BS) refers to pain associated with the occurrence of a lumbosacral transitional vertebra (LSTV). The global prevalence of low back pain is 18.3%^1^ and remains the most common musculoskeletal condition in the world. Point prevalence of low back pain in Africa is at 39%. However, in Uganda, the prevalence is at 20% and supersedes global prevalence^2^–^4^. The lumbosacral transitional vertebra is recognized when an elongated transverse process of the last lumbar vertebra fuses with the first sacral segment in variable degrees. This anomaly is recognized as a mechanical cause for lower back pain^5^ and is a common congenital anomaly, with reported prevalence varying between 3.3% and 35.6% with an average of 19.45%^6^. About 13% of all the patients with LSTV are asymptomatic. BS is diagnosed in about 4% - 8% of patients with low back pain of which 18.5% are under 30 years of age^7^. Studies in Nigeria showed the incidence of 37% in a hospital-based radiographic retrospective review. In comparison, a clinic-based Low Back Pain (LBP)- study showed a prevalence of 9.1% for Bertolotti syndrome with a male predominance^6^,^8^,^9^.

Patients commonly present with lower back pain, gluteal pain, S1 radiculopathy, and significant functional disability. Studies have shown that patients with LSTV and LBP have more pain and function impairment than patients without LSTV.^10^ Besides being underdiagnosed, the syndrome is difficult to treat. It also contributes to wrong-level surgery with potentially catastrophic consequences, especially when intraoperative imaging is not available for proper counting of vertebral segments. The miscount happens when there is complete sacralization or lumbarization.^10^–^13^.

Despite the high prevalence of LBP in Uganda, there is little literature about BS. This study sought to determine the prevalence of this syndrome, its clinical presentation, and associated functional disability. The findings will enhance clinician awareness to enable timely diagnosis and management.

## Methods

This was a descriptive cross-sectional study carried out at Mulago National Referral Hospital spine outpatient clinic for four months from November 2019 to February 2020. Approval for the study was by the Makerere University School of Medicine Research Ethics and Committee (SOMREC) and Mulago Hospital Research and Ethics Committee with reference #REC REF 2019-159.

Written informed consent was got from all and we followed good Clinical Practice guidelines. Using lumbar spine radiographs, we screened all consenting adult patients with chronic low back pain for LSTV. We classified Lumbosacral transitional vertebrae according to the Castellvi classification.

Exclusion criteria included all patients with LBP whose lumbar x-ray images showed fractures, tumors, infection, or other congenital anomalies of the lumbar spine that prevent study because of distortion of the anatomy and those who had undergone spine surgery.

### Sample size estimation

Using the Kish-Leslie formula to calculate sample size for means, taking a 95% confidence interval and error margin of 5% as the assumptions, we got a sample size of 389 patients.

### Sampling method

We consecutively recruited all participants with chronic low back pain who consented and had adequate lumbar spine radiographs into the study until the sample size was attained.

### Independent variables

They included socio-demographic characteristics, age, sex, and occupation.

### Dependent variables

These included:

- Clinical presentation of Bertolotti syndrome, including the severity of pain using the Visual analog score.
- Radiological classification according to Castellvi classification
- Functional disability using the Oswestry disability index.

### Study procedure

We consecutively screened 389 patients with chronic back pain for LSTV and identified 39 patients with BS using adequate lumbar radiographs done at MNRH using a digital x-ray machine, both lateral and anteroposterior views. The radiological classification of LSTV was according to Castellvi's classification^14^. We recorded the information from the radiographs in a data collection sheet. We consulted a radiologist for quality control and to ensure consistency in results.

We screened patients without LSTV out and didn't subject them to further examination or interviews.

We administered a questionnaire and did a physical examination for patients with BS. We inspected the patients for any deformities such as scoliosis and kyphosis. We palpated the lower back for muscle spasms and tenderness. We used the modified Schober's test to test for a range of motion of the lumbar spine.

Modified Schober's test involved marking the lumbar spine at the L5 and T12 spinous processes and the distance noted. Then the patients were requested to flex the lower back towards toe touch with knees straight. Then the distance was remeasured in that position using a tape measure. The test was deemed positive if the difference in measurements was less than 4 cm, showing limitation in lumbar flexion^15^.

We determined sacroiliac tenderness using the FABER, anterior gapping, and thigh thrust tests. We considered the patient to have sacral iliac tenderness if at least two of the tests were positive.

### Anterior gapping test

The patient was placed in the supine position. I applied downward pressure and outward to the anterior superior spines bilaterally. The test was deemed positive if the patient complained of pain in the posterior gluteal region.

### Thigh thrust test

With the patient still in the supine position, the hip was flexed and slightly adducted. Then, a posterior shearing force was applied through the femur onto the sacroiliac joint. Increasing pain showed a positive test.

### Faber

This test involved flexion, abduction, and external rotation of the femur at the hip joint. While holding down the anterior superior iliac spine on the opposite side, the pressure was applied at the medial side of the knee. This elicited tenderness if there was sacroiliac pathology.^16^

We then did a neurological exam to determine sensory and motor deficits. We examined dermatomes for touch and pain using cotton wool and pinpricks respectively comparing opposite dermatomes.^17^ Then muscle strength was tested for each nerve root to determine the myotome affected. The strength of the muscles acting across joints was determined. The nerve roots correspond to the following joint motions; L2 for hip flexion, L3 for knee extension, L4 for ankle dorsiflexion, L5 for toe extension, and S1 for ankle plantar flexion. The visual analog score was used for the assessment of the severity of pain. The Oswestry Disability Index (ODI) was used to determine the degree of functional disability (patient's perceived functional limitation). The Oswestry Disability Index (also known as the Oswestry Low Back Pain Disability Questionnaire) is a validated tool that many researchers and disability evaluators have used to measure a patient's permanent functional disability. This test is considered the ‘gold standard’ among other low back functional outcome tools and it has high reliability (R= 0.99).

It contains ten sections that access a patient's ability to live with back pain and the degree to which this pain is affecting daily life. Each section has scored a maximum of 5 if the last statement is marked. The total score is computed by converting it into a percentage that will grade disability from mildly impaired to crippled.^18^

The score calculated is interpreted as follows.

### Data analysis and management safety

We used STATA version 13 for descriptive statistics and summarized using means or standard deviation (SD) for normally distributed data, medians, the interquartile range for skewed data. The demographic characteristics are presented as frequencies and percentages.

Questionnaires and sample data extraction sheets were coded, assigned a unique identifier, and entered into excel after double verification of the entered data.

Radiographs photographed and daily backup kept on the cloud, PI's computer secured with a password, external drive, and Google drive. We stored completed questionnaires and sample data extraction sheets under lock and key at the study site. Back-up was performed daily.

## Results

A total of 39 out of 389 participants screened had Bertolotti syndrome. (10.1%) The majority were female 26/39 (66.7%). Those aged between 36 to 50 were 17/39 (43.6%) with a median age of 49 years. 14/39 (35.9%) were not employed. 12/39 (30.8%) patients had the age of onset of pain between 31 to 40 years.

The majority (95%) of patients reported axial pain with 66.7% of that being in the midline. We found radicular pain in 66.7% of patients and the majority (57.7%) had pain in the right lower limb. Almost half (45.5%) of patients had severe axial pain, while most patients had mild radicular pain (40.7%). Most patients had pain lasting over 19 hours of the day. (46.1%).

The majority (94.8%) had no lumbar spine deformity and the range of motion of the spine was limited in 71.8% of the patients according to a positive Schober's test. Some patients (23.1%) had muscle spasms on clinical examination.

In addition, most patients had tenderness in the midline of the lower lumbar spine at all the affected levels (mean 71.7%) but more especially at L5 (66.7%) while the right lower back was the next most reported site of tenderness at the S1 level (25.6%). Sacroiliac tenderness was more common on the right.

The commonest dermatome affected by sensory deficits was L5 on both sides and the least affected was L3. Also, 90% of patients had sensory deficits.

Then also 20% of patients had weakness in the lower limbs, especially the L5 nerve root in 20.8% of the patients. The next commonly affected nerve root was S1. Only 8% of patients had a severe weakness, with power less than 3/5.

Furthermore, 51.3% of patients had moderate disability according to the Oswestry disability index, with 12.8% being crippled by pain. Also, the commonest type of LSTV was type IIA, affecting 25.6% of the patients followed by type IIB and the least type was IIIA at 5.1%. Overall, type I has 25.6%, type II 46.1%, and type III with 25.6%; making type II the commonest type of LSTV in this study.

## Discussion

There is a paucity of information on the prevalence of Bertolotti syndrome in the world, especially in developing countries. Most of the data is from estimates of smaller hospital-based studies. In Sub- Saharan Africa, the prevalence of Bertolotti syndrome in the population is unknown. This could be because of the lack of African literature about it^19^. This study sought to bridge that knowledge gap.

This study has attempted to describe the clinical patterns of patients with BS and we found the prevalence to be 10.1%. This is higher than the global reported prevalence in other studies done in the general population at 5–7%.^20^,^21^. It is almost three times higher than that reported in some studies.^19^, ^21^.This high prevalence could be explained because MNRH is a referral center and thus the spine clinic receives a lot of refractory back pain cases. Females were most afflicted in this study, however, in similar studies, males were afflicted.^13^.^22^.^19^ We have attributed the findings to physiological and psychosocial differences in pain perception and pain reporting between males and females.^23^

Most patients were aged between 36 -50; with an average of 45.8 years. This is within the range of most studies 20,21. Thus, most patients began experiencing pain at an early age and continue to suffer for several more years. This is because BS is a congenital abnormality that is often clinically silent until the second or third decade^22^. Our patients may have taken longer to get a diagnosis since there is limited African literature and clinicians may not have considered it. All the patients interviewed revealed that it was the first time this radiological finding was pointed out to them. They had attended the spine clinic over several years and had not received such a diagnosis. It showed, therefore, that BS was unknown to the clinicians as well. Most patients in this study were unemployed. Nonmanual laborers were the next affected. Professionals were least affected. No studies have explored the relationship between Bertolotti syndrome and employment status or type of occupation. However, the findings correlate with studies that have explored relationships between occupation and back pain^24^. Patients reported failure to work or resume work because of the debilitating nature of the pain. Those without employment found it very difficult to do home chores. BS seems to have more long-lasting debility than other causes of mechanical back pain.

The majority in this study had tenderness in the axial spine, paravertebral region, buttock, and leg pain. This correlates with other studies in which low back axial pain was the most significant site of tenderness, followed by lumbar - buttock pain^22^,^25^,^26^. Half of the patients in this study had sacroiliac tenderness that was more common on the right. This was more than that reported in other studies^22^. Also, some studies attributed symptoms of buttock pain to sacroiliac disease^25^. The high prevalence of sacroiliac tenderness in the present study could also be because of the use of three different methods of evaluation, which could have increased the sensitivity to diagnose sacroiliac pain. This presentation could be because of alterations in the biomechanics of the lumbosacral junction due to the transitional vertebrae causing abnormal force transfers, joint overload of the sacroiliac joint, and muscle strain because of imbalances.

Radicular pain was found in the majority of patients, especially the right lower limb. This correlates with other studies which reported up to one-third of cases.^19^,^25^,^27^. For this study, the average age was higher and could explain more nerve root complaints associated with early disc degeneration. Most radiographs demonstrated early spondylosis, with intervertebral space narrowing, osteophytes, and sclerosis of endplates. This showed that patients with BS had earlier degeneration compared to other sufferers of back pain. Still, this is attributed to an alteration in the loading mechanisms of the region being altered. The side affected by the radiculopathy in the majority of patients correlated with the side in which an abnormal transverse process had an abnormal articulation or fusion. This was especially associated with type II and type III Castelli LSTV.

This study found that half of the patients had severe axial pain, with the majority having mild radicular pain. The average VAS for both radicular and axial pain was moderate pain. This compares to some studies.^26^ This shows that overall pain scores were worse in patients with Bertolotti syndrome than other low back pain causes. We attributed this to underdiagnosis and treatment. 10,25,28. As well, the anatomical sources of nerve compression are more in patients with BS such as disc prolapse causing both central, paracentral, and extra-foraminal compression besides the aberrant course of the nerves extra-osseous leads to compression especially when there is a pseudoarthrosis involved.

Most patients had pain lasting most of the day. This is comparable to findings by Morin et al. in which patients had pain most of the day and for over three years. 22, 25 The reason for this is unclear, but we postulate that most patients hadn't received a clear diagnosis and specific treatment. The pain generators in BS include discopathy, osteophytes, muscle strains and spasms, nerve root compression, sacroiliitis, pseudo-arthrosis, and extra-spinal nerve compression.

The present study showed that the majority had scoliosis on both examination and radiology. This agrees with several studies. Jain et al. reported that 100% had scoliosis. 20,29 We attribute this to changes in spine biomechanics because of LSTV.

We found that the range of motion of the spine was limited in some patients and most had no muscle spasms on clinical examination. This is similar to most studies 13. The limitation in the range of motion could be attributed to the pain caused by the structural alterations caused by LSTV. This study found fewer limitations and spasms compared with other studies. This could be because some patients had undergone physical therapy and had been on antispasmodics.

Almost all patients in this study had sensory deficits. The commonest dermatome affected by sensory deficits in this study was L5 on both sides and the least affected was L3. Weakness in the lower limbs was uncommon, affecting L5 and S1. This is similar to other studies.^30^, ^31^ ^26^ These findings can be attributed to early degeneration causing radiculopathy or extra-spinal nerve compression because of aberrant nerve course.

The study found that the clinical presentation of BS is like that of other causes of back pain except that it occurs in a younger age group, is more refractory to treatment. It needs a high index of suspicion to consider it in the differential diagnosis.

The key LSTV associated was type IIB.^32^,^33^,^34^ some studies have reported L6 and L4 symptoms in patients with type IIA Castelli LSTV.^27^,^35^,^36^. We thought this difference in nerve root affection to be caused by nerve roots in BS having altered function.^37^–^40^.

Many types of LSTV were found in the study, with type II the commonest. Also, the commonest subtype of LSTV was type IIA. These findings are comparable to other studies.^22^,^12^,^30^,^41^,^42^. Similar mechanisms of inheritance of LSTV, their expression, and penetrance can explain this.^6^,^43^. This is significant because type II is associated with a pseudoarthrosis between the transverse process of the last lumbar vertebra and the first sacral vertebra, causing a significant area of both osseous and nerve irritation. It also shows that type II is the most symptomatic type of LSTV.

Overall, in this study, most patients had moderate- to severe disabilities. This is comparable to the average disability found in other studies. These patients also had undergone more workups and had worse physical and mental health scores.^44^,^10^,^45^,^46^,^26^,^47^ In contrast howeve, Cynthia k. Peterson didn't find any correlation between LSTV presence and increased pain and disability scores, though older patients fared worse.^48^ The findings in the present study could be attributed to the fact that some patients had already undergone several conservative treatment modalities, including physical therapy. However, these are patients who have had such disability for several years such that the ODI taken doesn't show the full extent of disability. The patients with severe disability may represent patients whose pain is refractory to conservative management and needs further evaluation and more individualized treatment targeting key pain generators.

## Conclusion & Recommendations

Bertolotti Syndrome is a common condition that goes unrecognized, undiagnosed, untreated for long leading to severe functional disability. It affects a younger age group than other causes of back pain and presents with closely similar symptoms but with worse pain and disability.

We recommend that Bertolotti syndrome should be suspected and screened for among patients presenting with refractory low back pain.

Future research should explore pain generator identification using advanced imaging and finding specific treatment modalities for this condition.

Finally, a comparative study should be done between patients with BS and those without to determine differences in response to treatment.

## Figures and Tables

**Table:** Interpretation of scores

0% to 20%: minimal disability:	The patient can cope with most living activities. Usually, no treatment is indicated apart from lifting, sitting, and exercise advice.
21%–40%: moderate disability:	The patient experiences more pain and difficulty with sitting, lifting, and standing. Travel and social life are more difficult and they may be disabled from work. Personal care, sexual activity, and sleeping are not grossly affected and the patient can usually be managed by conservative means.
41%–60%: severe disability:	Pain remains the major problem in this group, but activities of daily living are affected. These patients require a detailed investigation.
61%–80%: crippled:	Back pain impinges on all aspects of the patient's life. Positive intervention is required.
81%–100%:	These patients are bed-bound or exaggerating their symptoms.

**Table 1:** Demographic characteristics of Bertolotti syndrome patients

Variable	Frequency	Percent
**Age**		
Median	49 (12)	
<36	7	17.9
36–50	17	43.6
>50	15	38.5
**Sex**		
Male	13	33.3
Female	26	66.7
**Occupation**		
Manual labor	8	20.5
Non-Manual labor	12	30.8
professional work	5	12.8
No occupation	14	35.9
**Age at Pain onset**		
<18	1	2.6
18–30	11	28.2
31–40	12	30.8
41–50	11	28.2
>50	4	10.3

**Table 2:** Pain characteristics

Variable	Frequency	Percent
Axial pain		
Yes	39	100
No	0	0
Axial pain site		
Left lower back	4	10.3
Right lower back	7	17.9
Midline	26	66.7
Left buttock	0	0.0
Right buttock	2	5.1
Radicular pain		
Yes	26	66.7
No	13	33.3
Radicular pain site(n=26)		
Left lower limb	11	42.3
Right lower limb	15	57.7
VAS-Axial pain		
Mean	6.3	
Mild	7	18.0
Moderate	13	33.3
Severe	19	48.7
VAS-Radicular Pain(n=26)		
Mean	6.2	
Mild	11	40.7
Moderate	5	18.5
Severe	10	38.5
Pain duration(hours)		
<5	15	38.5
6–12	7	17.9
>19	18	46.1
Schober's test		
Negative	28	71.8
Positive	11	28.2
Sacroiliac tenderness		
Right	8	20.5
Left	7	17.9
Both	4	10.2
None	20	51.2

**Table 3:** Functional disability and type of LSTV

Variable	Frequency	Percent
**Oswestry disability index**		
Mild	6	15.4
Moderate	20	51.3
Severe	8	20.5
Crippled	5	12.8
**Castellvi classification**		
IA	5	12.8
1B	5	12.8
IIA	10	25.6
IIB	8	20.5
IIIA	2	5.1
IIIB	5	12.8
IV	4	10.3

## Data Availability

The datasets used and/or analysed during the current study are available from the corresponding author on request.

## List Of Abbreviations

AP: Anteroposterior
BS: Bertolotti Syndrome
LAT: Lateral View
LBP: Low Back Pain
LATV: Lumbosacral Transitional Vertebrae
MNRH: Mulago National Referral Hospital
ODI: Oswestry Disability Index
VAS: Visual Analogue Scale
PI: Principal investigator
